# Carbon-ion Radiotherapy for Isolated Lymph Node Metastasis After Surgery or Radiotherapy for Lung Cancer

**DOI:** 10.3389/fonc.2019.00731

**Published:** 2019-08-07

**Authors:** Katsuyuki Shirai, Yoshiki Kubota, Tatsuya Ohno, Jun-ichi Saitoh, Takanori Abe, Tatsuji Mizukami, Yasumasa Mori, Hidemasa Kawamura, Keiko Akahane, Takashi Nakano

**Affiliations:** ^1^Department of Radiology, Saitama Medical Center, Jichi Medical University, Saitama, Japan; ^2^Gunma University Heavy Ion Medical Center, Maebashi, Japan; ^3^Department of Radiation Oncology, Faculty of Medicine, University of Toyama, Toyama, Japan

**Keywords:** lymph node metastasis, carbon-ion radiotherapy, particle beam therapy, non-small cell lung cancer, postoperative recurrence

## Abstract

**Purpose:** Mediastinal and hilar lymph node metastasis is one of the recurrence patterns after definitive treatment of lung cancer. Salvage radiotherapy (RT) can be a treatment option for lymph node metastasis. However, the usefulness of additional RT remains unclear after surgery or RT for the primary lung tumor. We retrospectively evaluated the efficacy and safety of hypofractionated carbon-ion RT for isolated lymph node metastasis.

**Methods and Materials:** Between April 2013 and August 2016, 15 consecutive patients with isolated lymph node metastasis underwent carbon-ion RT. The pretreatment evaluations confirmed the isolated lymph node metastasis and the absence of local recurrence or distant metastasis, which was oligometastatic disease. The median age was 72 (range, 51–83) years, with 11 male patients. The first treatments for primary lung tumors were carbon-ion RT for 8 patients and surgery for 7 patients. There were 9 adenocarcinomas, 4 squamous cell carcinomas, 1 adenosquamous cell carcinoma, and 1 mucoepidermoid carcinoma. Most patients (93%) were irradiated with 52.8 Gy relative biological effectiveness in 12 fractions for 3 weeks. There were no patients treated with concurrent or adjuvant therapy such as chemotherapy, molecular-targeted therapy, or immunotherapy. Adverse events were evaluated according to the Common Terminology Criteria for Adverse Events (version 4.0).

**Results:** The median follow-up for surviving patients was 28 months. One patient experienced local lymph node recurrence, and the 2-year local control rate was 92% for all patients. Distant metastasis was observed in 7 patients, and 2-year progression-free survival rate was 47%. During follow-up, there were 4 deaths from lung cancer, and the 2-year overall survival rate was 75%. There were 2 patients with acute grade 2 esophagitis and 2 with late grade 2 cough, which were improved by conservative therapy. There were no other grade 2 or higher adverse events.

**Conclusions:** Hypofractionated carbon-ion RT showed excellent local control and overall survival without severe toxicities in lung cancer patients with isolated lymph node metastasis after surgery or carbon-ion RT for primary lung tumors. A multi-institutional prospective study is required to establish the efficacy and safety of carbon-ion RT.

## Introduction

Mediastinal and hilar lymph node metastasis is one of the recurrence patterns after definitive treatment of lung cancer. After radiotherapy (RT) for early stage lung cancer, lymph node metastasis developed in 7.6–8.6% of cases (1, 2). Japan Clinical Oncology Group Study (JCOG0403) showed the detail of patterns of failures after stereotactic body RT for stage I lung cancer, and the lymph node metastasis was observed in 14.2% and isolated lymph node metastasis without other recurrent site was 3.0% (3). Hilar and mediastinal lymph node metastasis after surgery was detected in 7% of stage I to II lung cancer patients (4). Pathological N1 and N2 were incidentally observed in 9.3 and 12.3% in clinical stage IA lung cancer patients who underwent pulmonary resection (5). The ideal treatment strategy for isolated lymph node metastasis remains uncertain. Lymph node metastasis can accompany distant metastasis, and chemotherapy is often performed. On the other hand, isolated lymph node metastasis can be curable by aggressive local treatment including surgery and RT, because the disease site is only regional. Surgery is the mainstay of isolated lymph node metastasis, but salvage surgery after the first surgery is not feasible due to patient burden. Salvage RT can be a treatment option for lymph node metastasis, and the establishment of the standard RT method is necessary.

Carbon-ion RT is a radical non-surgical treatment for achieving a high local control rate without severe adverse events. Carbon-ion has good dose-localization because of the Bragg peak, so the radiation dose can be minimized to the surrounding normal tissue (6). Moreover, a carbon-ion beam offers high biological effectiveness, which results in favorable tumor control. Previously, the safety and efficacy of carbon-ion RT were reported for early stage and locally advanced lung cancer (7, 8). Moreover, a few retrospective studies demonstrated the usefulness of carbon-ion RT for isolated lymph node recurrence of esophageal cancer, colorectal cancer, and several tumors (9–11). Therefore, carbon-ion RT might be a promising treatment for lung cancer patients with isolated lymph node metastasis after initial treatments. In this study, we retrospectively evaluated the safety and efficacy of hypofractionated carbon-ion RT for isolated lymph node metastasis in lung cancer.

## Methods and Materials

### Patients

Between April 2013 and August 2016, 15 consecutive patients with isolated lymph node metastasis were treated with passive irradiation carbon-ion RT at the Gunma University Heavy Ion Medical Center. The clinical characteristics are shown in Table 1. The median age was 72 (range, 51–83) years, with 11 male patients. The first treatments for primary lung tumors were carbon-ion RT for 8 patients (post-carbon-ion RT group) and surgery for 7 patients (post-surgery group). In post-carbon-ion RT group, there were 6 patients with stage I and 2 with stage II without lymph node metastasis. The dose of carbon-ion RT was expressed as “Gy (relative biological effectiveness [RBE]).” Three patients with smaller tumors than 30 mm were treated with carbon-ion RT of 52.8 Gy (RBE) in 4 fractions, and 5 patients with larger tumors than 30 mm were treated with 60.0 Gy (RBE) in 4 fractions. There were 5 patients with stage I, and 2 with stage III in post-surgery group. There were 5 patients received lobectomy with a mediastinal lymph-node dissection and 2 patients received limited resection (segmentectomy and wedge resection). One patient had tegafur-uracil as postoperative chemotherapy. Another patient in post-surgery group received carboplatin and pemetrexed for lymph node recurrence, and the response was stable disease. There were 9 adenocarcinomas, 4 squamous cell carcinomas, 1 adenosquamous cell carcinoma, and 1 mucoepidermoid carcinoma. The pretreatment evaluations included a physical examination, computed tomography, brain magnetic resonance imaging, and 18-fluorodeoxyglucose-positron emission tomography. These evaluations confirmed the isolated lymph node metastasis and the absence of local recurrence or distant metastasis, which was oligometastatic disease.

**Table 1:** Patient and tumor characteristics.

**Characteristics**		**Total** **(*n* = 15)**	**Post-carbon-ion** **RT group** **(*n* = 8)**	**Post-surgery group** **(*n* = 7)**
Age, Median (years)		72 (range, 51–83)	73 (range, 54–82)	67 (range, 48–83)
Sex	Male	11	6	5
	Female	4	2	2
Histology	Adenocarcinoma	9	4	5
	Squamous cell carcinoma	4	4	0
	Adenosquamous cell carcinoma	1	0	1
	Mucoepidermoid carcinoma	1	0	1
Radiation dose	52.8 Gy (RBE) in 12 fractions	14	7	7
	48.0 Gy (RBE) in 12 fractions	1	1	0
Stage at the initial treatment	Stage I	11	6	5
	Stage II	2	2	0
	Stage III	2	0	2
Type of surgery	Lobectomy	−	−	5
	Segmentectomy	−	−	1
	Wedge resection	−	−	1

*RT, Radiotherapy*.

### Treatment

All patients provided written informed consent before treatment. The detailed techniques for carbon-ion RT and treatment planning have been reported previously (7, 8, 12). A customized patient pillow (Moldcare; Alcare, Japan) and a body shell (Shellfitter; Kuraray, Japan) were used to immobilize the patients. While patient's respiration was monitored with a respiratory gating system (AZ-733; Anzai Medical, Japan), CT scan was performed around the peak exhalation for treatment planning with 2 mm of CT slice thickness. Treatment was performed by respiratory-gated irradiation technique, and the adopted gating level of respiratory motion was 30% level of the wave amplitude around peak exhalation. The clinical target volume (CTV) included all swollen lymph nodes and potentially microscopic disease. CTV1 included 5 mm margin around gross tumor volume (GTV) and the lymph node station. CTV2 included only 5 mm margin around GTV. Involved filed RT was performed without elective regional lymph node irradiation. The planning target volume (PTV) included a 3–5 mm margin around the CTV to account for setup error and respiratory motion. The median PTV1 volume was 177 ml (range, 38–264 ml) and the median PTV2 volume was 43 ml (range, 18–86 ml), respectively. Median beam number was 3 (range, 3–4). A median total dose was 52.8 Gy (RBE) (range, 48.0–52.8 Gy [RBE]) in 12 fractions for 3 weeks with a dose fraction of 4.0–4.4 Gy (RBE). The safety and efficacy of the dose fraction regimen has been shown in the previous studies for para-aortic lymph node metastasis from colorectal cancer and for postoperative lymph node metastasis of esophageal cancer (9, 10). Median interval time between the first and second treatments was 16 months (range, 6–72 months). There were no patients treated with concurrent or adjuvant therapy such as chemotherapy, molecular-targeted therapy, or immunotherapy. Dose restriction of spinal cord was suppressed maximum dose <30 Gy (RBE), and the percentage of the lung volume receiving 20 Gy (RBE) was <20%. Although the dose constraints for esophagus, trachea, and bronchus, were not clearly established in carbon-ion RT treatment, the volumes of 50 Gy (RBE) were suppressed as low as possible based on our institutional policy.

### Evaluation

Local control, progression-free survival, and overall survival rates were calculated from the start of carbon-ion RT for lymph node metastasis to each event by the Kaplan-Meier method. Acute and late adverse events were evaluated according to the Common Terminology Criteria for Adverse Events (version 4.0). This retrospective study was reviewed and approved by our Institutional Review Board (160030). Dose distributions of the first and second carbon-ion RT were accumulated using deformable image registration, and a dose-volume histogram (DVH) analysis was performed using MIM Maestro (Version 6.0.2; MIM Software Inc., OH, USA). Percent and actual volumes for organs at risk were shown as a mean ± standard deviation for all patients. Differences between two groups were compared by Student's *t*-test, and a *p* < 0.05 was considered statistically significant. Statistical analyses were performed using SPSS software (Version 25.0; SPSS Inc., Chicago, IL, USA). The biologically equivalent doses in 2-Gy fractions (EQD_2_) were converted based on an alpha/beta ratio of 3.0 Gy (13).

## Results

### Clinical Outcomes

**Figure 1:**
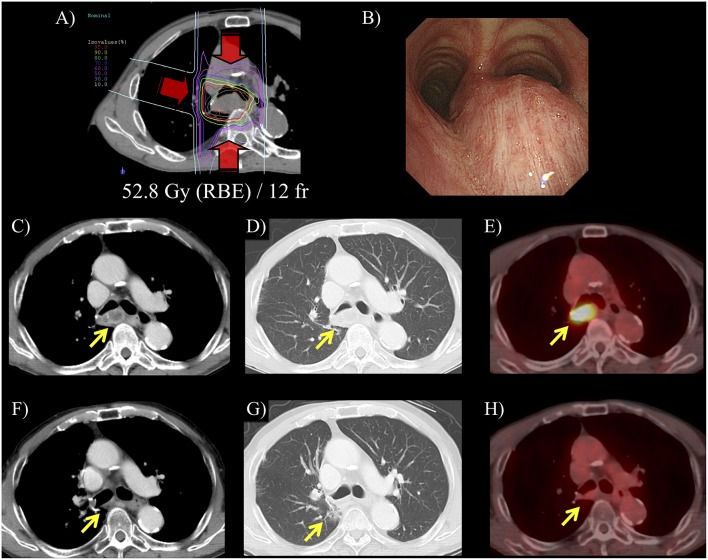
A representative case of carbon-ion radiotherapy (RT) for postoperative isolated lymph node metastasis. Patient was a 72-year-old male and mediastinal lymph node metastasis was detected at 2-year after surgery for primary lung tumor. **(A)** Hypofractionated carbon-ion RT was performed for the lymph node metastasis using 52.8 Gy (RBE) in 12 fractions for 3 weeks. **(B)** Before carbon-ion RT, bronchoscope revealed an extrinsic mass effect in the trachea. **(C,D)** CT showed that isolated mediastinal lymph node metastasis was 35 mm. **(E)** 18-fluorodeoxyglucose-positron emission tomography (FDG-PET) showed that maximum standardized uptake value was 7.6. in this lesion. **(F)** After carbon-ion RT, CT showed that the lymph node metastasis was diminished. **(G)** Pulmonary window image showed limited radiation fibrosis at the irradiated area. **(H)** FDG-accumulation in the lymph node was diminished and this patient had 2-years disease-free survival after the carbon-ion RT.

**Figure 2:**
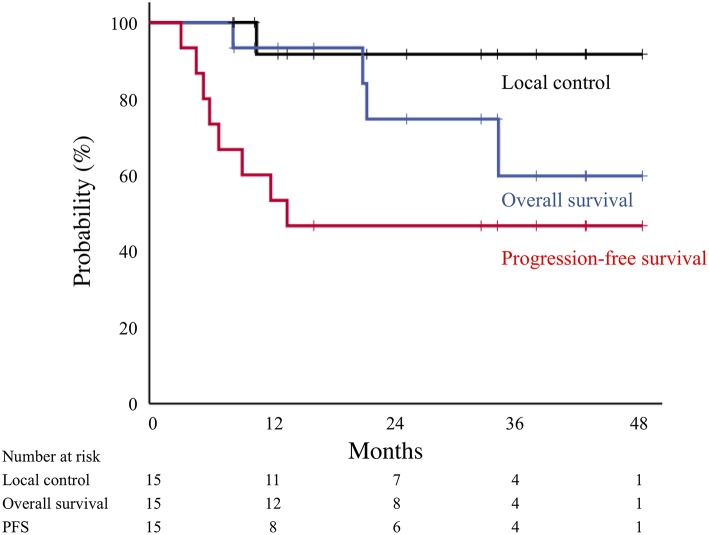
Local control (black line), overall survival (blue line), and progression-free survival (red line) curves for all patients treated by carbon-ion radiotherapy.

The median follow-up for surviving patients was 28 months (range, 8–48 months). Representative cases of post-surgery and post-carbon-ion RT groups are showed in Figure 1 and Supplementary Figure 1. Of the 15 patients, 1 patient (7%) experienced local lymph node recurrence. The patient had primary lung tumor recurrence at the same time, and salvage surgery performed. The 2-year local control rate was 92% (Figure 2). There were two patients with lymph node metastasis outside the radiation field, who had simultaneously distant metastasis. There was no isolated lymph node metastasis without distant metastasis after carbon-ion RT. Distant metastasis was observed in 7 patients (brain, *n* = 2; liver, *n* = 1; adrenal gland, *n* = 1; bone, *n* = 1; lung, *n* = 1, pleural dissemination, *n* = 1). Two patients with brain metastasis underwent stereotactic RT and 1 of them remained alive without disease after treatment. One patient with adrenal gland metastasis received RT. There were two patients with EGFR mutations received tyrosine kinase inhibitors. Two patients without EGFR mutations received chemotherapy. There were no patients received immunotherapy in this study. The 2-year progression-free survival rate was 47%. During follow-up, there were 4 deaths from lung cancer and the 2-year overall survival rate was 75%. There were no deaths due to other diseases or adverse events. According to a stage at the initial treatment, the 2-year overall survival rates of stage I (*n* = 11), II (*n* = 2), and III (*n* = 2) were 60, 100, and 100%, respectively. For the post-carbon-ion RT group (*n* = 8), the local control, progression-free survival and overall survival rates at 2 years were 86, 50, and 71%, respectively. These rates were 100, 43, and 86% for the post-surgery group (*n* = 7), respectively.

### Adverse Events

Acute and late adverse events of all patients are summarized in Table 2. Acute grade 2 esophagitis was observed in 2 patients (13%), which improved after completion of irradiation. There were no other severe acute adverse events, such as pneumonitis, dermatitis, or chest pain. Late grade 2 cough was temporarily observed in 2 patients (13%), which improved by conservative therapy. There were no radiation pneumonitis grade 2 or higher adverse events in the post-carbon-ion RT and post-surgery groups. Furthermore, there were no rib fractures, bronchial stenosis, or atelectasis.

**Table 2:** Acute and late adverse events of all patients (*n* = 15).

**Acute adverse events**	**Grade 2**	**Grade 3**	**Grade 4**
Pneumonitis	0 (0%)	0 (0%)	0 (0%)
Dermatitis	0 (0%)	0 (0%)	0 (0%)
Chest wall pain	0 (0%)	0 (0%)	0 (0%)
Esophagitis	2 (13%)	0 (0%)	0 (0%)
**Late adverse events**	**Grade 2**	**Grade 3**	**Grade 4**
Pneumonitis	0 (0%)	0 (0%)	0 (0%)
Dermatitis	0 (0%)	0 (0%)	0 (0%)
Chest wall pain	0 (0%)	0 (0%)	0 (0%)
Esophagitis	0 (0%)	0 (0%)	0 (0%)
Cough	2 (13%)	0 (0%)	0 (0%)

### Dose-Volume Histogram Analysis

**Figure 3:**
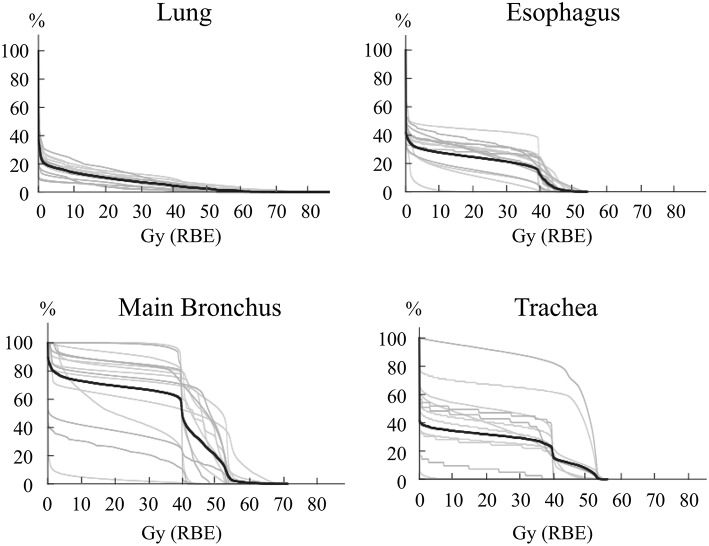
Dose-volume histogram for the esophagus, lung, main bronchus, and trachea. The lines representing each patient are in gray, and the average is a black line. For patients that received carbon-ion radiotherapy (RT) as the first treatment, these histograms were composed of both the first and second RT.

The percentage of the lung volume receiving 20 Gy (RBE) of carbon-ion RT (V20) for lymph node metastasis was 7.3 ± 3.9% (range, 2.7–17.3%) for all patients. In composite treatment planning including first RT and lymph node RT, the lung V20 was 10.0 ± 4.0% (range, 3.8–17.3%) (Figure 3). These values for post-carbon-ion RT and post-surgery groups were 11.6 ± 2.3% (range, 9.3–15.2%) and 8.1 ± 4.8% (range, 3.8–17.3%), respectively. Because the dose fractionation was different between first RT and lymph node RT, EQD_2_ were calculated based on an alpha/beta ratio of 3.0 Gy (Supplementary Figure 2). In composite treatment planning, the lung V20 (EQD_2_) was 10.4 ± 4.1% (range, 3.6–16.8%). These values for post-carbon-ion RT and post-surgery groups were 12.4 ± 2.1% (range, 9.9–15.2%) and 8.2 ± 4.9% (range, 3.6–16.8%), respectively. The percentage volume of 50 Gy (RBE) for the esophagus, main bronchus, and trachea was 0.4 ± 0.9% (range, 0–2.9%), 19.7 ± 19.1% (range, 0–54.9%), and 7.0 ± 14.5% (range, 0–48.7%), respectively. The actual volume of 50 Gy (RBE) for the esophagus, main bronchus, and trachea was 0.2 ± 0.4 ml (range, 0–1.3 ml), 3.7 ± 3.8 ml (range, 0–11.5 ml), and 1.9 ± 3.6 ml (range, 0–10.8 ml), respectively. The dose of 2 ml for the esophagus, main bronchus, and trachea were 36.6 ± 14.7 Gy (RBE) (range, 0.1–48.3), 45.7 ± 14.0 Gy (RBE) (range, 0.8–60.0), and 31.7 ± 21.8 Gy (RBE) (range, 0–52.7), respectively.

The esophageal dose was compared between the patients with acute esophagitis grade 1–2 and grade 0 (Supplementary Table 1 and Supplementary Figure 3). The patients with grade 1–2 had significantly higher V20 and V30 Gy (RBE), and mean dose of esophagus than those with grade 0.

## Discussion

Hypofractionated carbon-ion RT showed excellent local control and overall survival without severe toxicities in lung cancer patients with isolated lymph node metastasis. Previously, several reports showed the outcomes of photon for loco-regional recurrence, including as postoperative site recurrence, a second lung tumor, and/or lymph node recurrence (14–16). However, these recurrent diseases should be evaluated separately because the treatment strategy is different for each disease. To date, there have been no prospective studies to evaluate RT for only lymph node recurrence of lung cancer, and the role of RT on these diseases remains unclear. A few retrospective studies have been reported, which are summarized in Table 3 (17–20).

**Table 3:** Retrospective studies of irradiation to the hilar and mediastinal lymph node recurrence in lung cancer.

**References**	**Type of Salvage RT for lymph node Median RT dose**	**Stage at initial treatment I/II/III**	**First treatment for primary lung tumor**	**Local control**	**Overall survival**	**Pneumonitis grade ≥ 2**	**Lung V20 for lymph node RT**	**Composite Lung V20**
Kilburn et al. (17)	Photon (IFRT) 66 Gy / 33 fr.	10/2/0	Photon (*n* = 12)	100% (2 y)	29% (2 y)	17%	−	25% (13–29%)
Ward et al. (18)	Photon (IFRT) 45 Gy / 15 fr.	12/2/1	Photon (*n* = 15)	84% (1 y)	73% (1 y)	−	−	−
Manabe et al. (19)	Photon (Elective regional irradiation) 60 Gy / 30 fr.	27/0/0	Photon (*n* = 14) Surgery (*n* = 13) All (*n* = 27)	58% (5 y) 92% (5 y) 81% (5 y)	14% (5 y) 36% (5 y) 24% (5 y)	43% 31% 37%	14% (3–40%) 15% (8–37%) 15% (31–39%)	−−−
Seol et al. (20)	Photon (IFRT) 66 Gy / 33 fr.	17/7/7	Surgery (*n* = 31)	76% (2 y)	58% (2 y)	16%	−	−
Present study	Carbon-ion RT (IFRT) 52.8 Gy (RBE) / 12 fr.	11/2/2	Carbon-ion RT (*n* = 8) Surgery (*n* = 7) All (*n* = 15)	86% (2 y) 100% (2 y) 92% (2 y)	71% (2 y) 86% (2 y) 75% (2 y)	0% 0% 0%	6% (2–13%) 8% (4–17%) 6% (2–17%) EQD_2_ data	12% (10–15%) 8% (4–17%) 10% (4–17%) EQD_2_ data

*RT, Radiotherapy; y, year; V20, percentage of the lung volume receiving 20 Gy; IFRT, Involved field radiotherapy*.

Our study using carbon-ion RT reported that the 2-year local control and overall survival rates were 92 and 75%, respectively. Previous studies using photon therapy also showed a 76–100% local control rate at 1–2 years (17, 18, 20). Manabe et al. reported long term outcomes with an 81% local control rate for 5-years (19). These results indicated that local control rate associated with carbon-ion RT was as high as those of photon therapy. However, the overall survival rates indicated in photon therapy studies were between 29 and 58% at 2-years (17, 20), which were relatively poorer than 75% of our results. It is difficult to compare the overall survivals in these studies, because the initial treatments and stages are different (Table 3). However, the difference in overall survival between these studies may be related to different adverse events rates between photon therapy and carbon-ion RT. Grade 2 or higher radiation pneumonitis rates were 16–43% for photon therapy in previous studies (4, 19, 20), while we showed that the pneumonitis rate was 0% for carbon-ion RT (Table 3). Generally, radiation pneumonitis decreases a patient's performance status and quality of life, which can be related to a worse long-term prognosis. Especially, repeated RT for lymph node recurrence after primary lung tumors should be carefully performed, because irradiation areas are wide and an excess radiation dose can be caused by overlapping RT. Manabe et al. reported that grade 2 or higher radiation pneumonitis was 43% in the post-RT group which was relatively higher than 31% of the post-surgery group (19). Moreover, grade 5 radiation pneumonitis was observed in 1 patient in the post-RT group. They were concerned that the first RT for the primary lung tumors would be a risk for adverse events when lymph node disease was irradiated, compared with the surgery for the first treatment. On the other hand, our study using carbon-ion RT showed that severe pneumonitis rates were 0% in the post-carbon-ion RT and post-surgery groups. These findings indicate that carbon-ion RT is a safe radiation technique even if the treatment is repeated, compared with photon therapy.

The difference in the pneumonitis rate between photon therapy and carbon-ion RT was due to high conformality of carbon-ion RT compared with photon therapy. Furthermore, we performed involved filed RT without elective regional lymph node irradiation, which may contribute to the less radiation pneumonitis rate. Kilburn's study of photon therapy showed that the composite lung V20 of first and second RT was 25% (range, 13–29%) (17), while the lung V20 in our study using carbon-ion RT was 10.4 ± 4.1% (range, 3.6–16.8%) in EQD_2_. The value of lung V20 of carbon-ion RT was approximately half of that of photon therapy for lymph node metastasis, which is consistent with a previous study of DVH parameters for stage I lung cancer (21). Kilburn's study showed that 17% experienced grade 2 or higher pneumonitis and 8% experienced grade 3 (17). Repeated photon therapy was associated with a high value for lung V20, which may result in severe pneumonitis. On the other hand, carbon-ion RT was not associated with grade 2 or higher pneumonitis due to the low value of lung V20.

Hilar and mediastinal lymph node metastases are frequently located close to organs at risk. Therefore, it is important to establish the treatment planning methods to avoid the organs in carbon-ion RT, as well as in photon therapy (22, 23). Previously, a few studies reported that carbon-ion RT irradiation to the lymph node area has been safely performed for locally advanced lung cancer (24, 25). This study used the dose constraint of 50 Gy (RBE) for esophagus (25). In our study, the volumes of 50 Gy (RBE) for the esophagus, trachea, and bronchus, are suppressed as low as possible based on our treatment policies. However, the dose constraints of carbon-ion RT for mediastinal organs were not clearly defined. Therefore, we carefully evaluated the actual dose of 50 Gy (RBE) for these organs and these adverse events. For example, the average actual volume of 50 Gy (RBE) for the esophagus in all patients was 0.2 ml, and acute temporal esophagitis of grade 2 was 13% without severe esophageal ulcer or stenosis. This adverse event rate associated with carbon-ion RT was considered less than other photon therapy of 33% with esophagitis grade 2 (18). We did not perform elective regional lymph node irradiation, which may contribute to the less adverse events. Severe adverse events such as esophageal ulcers and tracheal fistulas were not observed in this study. Furthermore, the incidence of common adverse events was also less in our study. These results indicated that our treatment policies can be reference data for safe treatment planning in delivery of carbon-ion RT. And, our study showed that symptomatic esophagitis (grade 1–2) was significantly associated with high esophageal dose. However, number of our patients was too small to establish the dose constraints, and further studies are required to determine the clear cut off value by using a DVH analysis.

In our study, 2-year progression-free survival was 47% because distant metastasis often developed. One possible reason for the high metastasis rate was the presence of micrometastatic disease at the time of lymph node recurrence. Concurrent and/or adjuvant chemotherapy could help eradicate micrometastatic disease and suppress the distant metastasis. However, there is a lack of evidence on the role of combination of chemotherapy for isolated lymph node metastasis. Seol et al. reported that addition of chemotherapy to RT did not improve the survival for these patients (20). Recently, adjuvant treatment using the immune checkpoint inhibitor, durvalumab, was reported to improve the prognosis for locally advanced lung cancer patients (26, 27). In this study, there were no evaluation of PD-L1 expression and immune-check point inhibitor was not performed. The use of this therapy in combination with carbon-ion RT is expected to improve the outcomes of locally advanced disease such as lymph node metastasis.

Considering the progression-free survival was poor, overall survival was favorable in our study. As described above, grate local control and minimum toxicities of carbon-ion RT contributed to the survival benefit. Furthermore, post-treatment after distant metastasis may improve the survival, because all patients (*n* = 7) with distant metastasis in our study received any treatments including RT (*n* = 3), tyrosine kinase inhibitors (*n* = 2), or chemotherapy (*n* = 2). Recently, molecular-based personalized therapy has highly developed in lung cancer field, and tyrosine kinase inhibitor is more effective than chemotherapy for patient with EGFR mutations (28). Also, consolidative RT for a limited number of distant sites has been reported to extend progression disease and delay the appearance of new lesion in stage IV lung cancer patients (29). Even if distant metastasis is observed, aggressive treatments based on the patient's condition have a potential to contribute the survival.

One of the merits of carbon-ion RT is shorter treatment duration than conventional RT using photon therapy. However, longer-term follow-up is required to evaluate the efficacy and safety because different dose fraction from conventional RT was used. The treatment duration of carbon-ion RT can be shortened, because the high linear energy transfer beam has unique physical and biological properties (30). Generally, the radical RT schedule of photon therapy is considered 60–66 Gy in 2 Gy dose fractions for 6 weeks. Previous studies of photon therapy for lymph node irradiation used these radiation dose fractionation schedules (17, 19). However, our study of hypofractionated carbon-ion RT used 52.8 Gy (RBE) in 12 fractions for 3 weeks. It is important to decrease a patient's burden by using a short treatment duration especially in a recurrent disease setting.

The present study has a few limitations, including the retrospective study design, the small number of patients, and the single-center setting. A multi-institutional prospective study for isolated lymph node metastasis in lung cancer is required to establish the efficacy and safety of carbon-ion RT.

To conclude, hypofractionated carbon-ion RT showed excellent local control and overall survival without severe toxicities in lung cancer patients with isolated lymph node metastasis. Carbon-ion RT is considered an option for salvage treatment of isolated lymph node metastasis.

## Data Availability

All datasets generated for this study are included in the manuscript.

## Ethics Statement

This study was reviewed and approved by our Institutional Review Board (160030). The protocol was performed in accordance with the Declaration of Helsinki and all patients provided their informed consent.

## Author Contributions

KS, YK, TO, and JS designed and directed the analysis. KS, TA, and TM generated a database and performed data collection. KS, YM, HK, and KA contributed to the analysis of the results and performed the statistical analysis. TO and TN supervised the project.

### Conflict of Interest Statement

The authors declare that the research was conducted in the absence of any commercial or financial relationships that could be construed as a potential conflict of interest.

## Supplementary Material

Supplementary Figure 1 A representative case of carbon-ion radiotherapy (RT) for isolated lymph node metastasis in post-carbon-ion RT group. **(A)** Carbon-ion RT was performed as the first treatment on an 82-year-old male patient with stage I lung cancer using 52.8 Gy (RBE) in 4 fractions for 1 week. Mediastinal lymph node metastasis was detected 1-year post-treatment. **(B)** Hypofractionated carbon-ion RT was performed for the lymph node recurrence using 52.8 Gy (RBE) in 12 fractions for 3 weeks. **(C)** 18-fluorodeoxyglucose-accumulation in the lymph node was diminished and this patient had 3-years disease-free survival after the second treatment. **(D)** A dose-volume histogram of this patient comprised of the first and second carbon-ion RT. **(E)** A dose-volume histogram of this patient comprised of the first and second carbon-ion RT by using EQD_2_ conversion.

Supplementary Figure 2 Dose-volume histogram for the esophagus, lung, main bronchus, and trachea by using EQD_2_ conversion. The lines representing each patient are in gray, and the average is a black line. For patients that received carbon-ion radiotherapy (RT) as the first treatment, these histograms were composed of both the first and second RT.

Supplementary Figure 3 **(A)** The dose-volume histogram parameters of 10 patients with acute esophagitis grade 1–2 (red line) and of 5 patients with grade 0 (blue line). Bold red and blue lines showed the average parameters of acute esophagitis grade 1–2 and grade 0, respectively. **(B)** The dose-volume histogram parameters were calculated by using EQD_2_ conversion.

Supplementary Table 1 A comparison of esophageal dose between acute esophagitis grade 0 and Grade 1–2.
