# Supplementary figures and images for: Carbon-ion Radiotherapy for Isolated Lymph Node Metastasis After Surgery or Radiotherapy for Lung Cancer
Source: Front Oncol. 2019 Aug 7;9:731. doi: 10.3389/fonc.2019.00731 (PMC6692658; doi:10.3389/fonc.2019.00731)

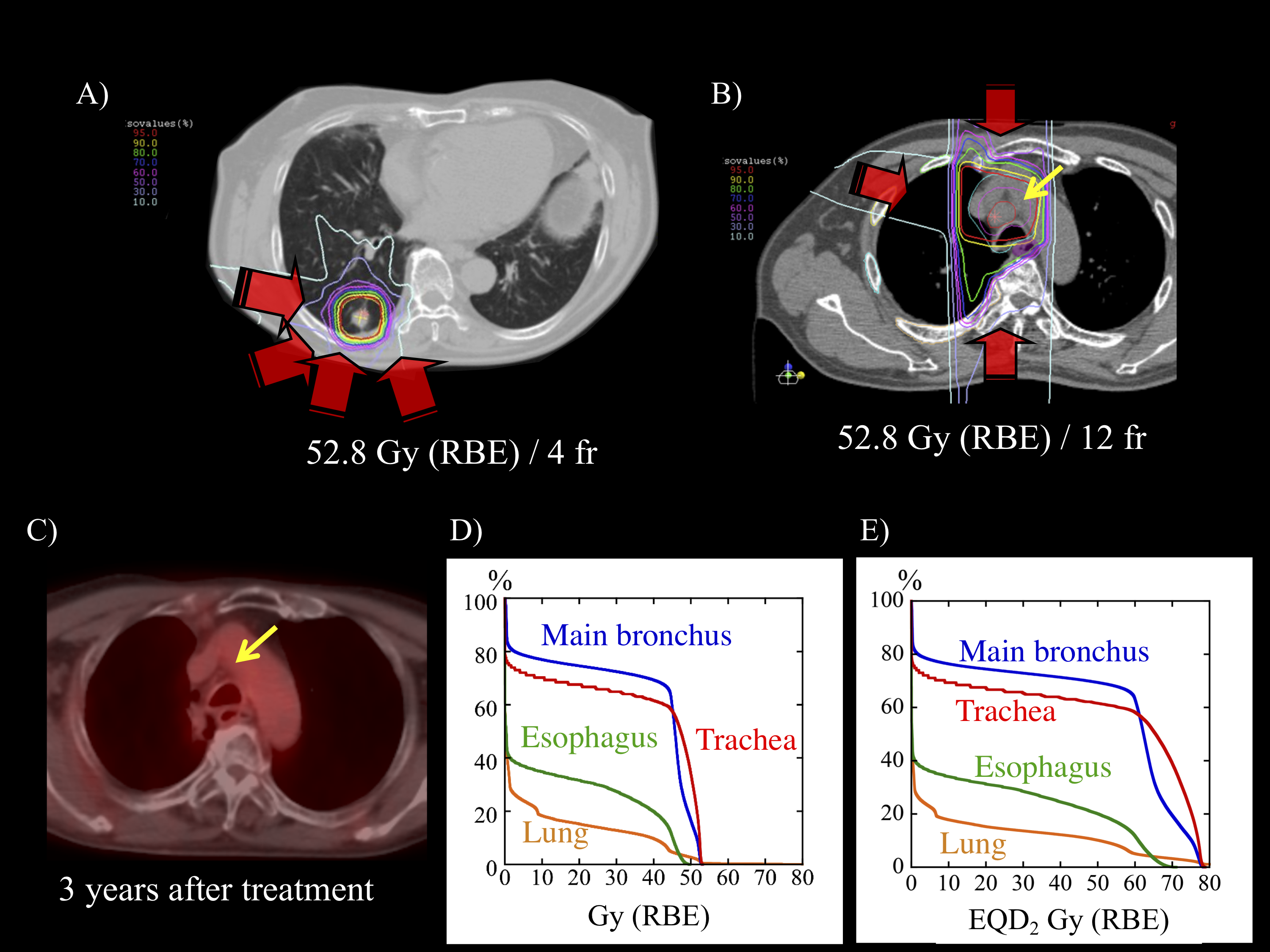

Supplement: Supplementary Figure 1 — A representative case of carbon-ion radiotherapy (RT) for isolated lymph node metastasis in post-carbon-ion RT group. (A) Carbon-ion RT was performed as the first treatment on an 82-year-old male patient with stage I lung cancer using 52.8 Gy (RBE) in 4 fractions for 1 week. Mediastinal lymph node metastasis was detected 1-year post-treatment. (B) Hypofractionated carbon-ion RT was performed for the lymph node recurrence using 52.8 Gy (RBE) in 12 fractions for 3 weeks. (C) 18-fluorodeoxyglucose-accumulation in the lymph node was diminished and this patient had 3-years disease-free survival after the second treatment. (D) A dose-volume histogram of this patient comprised of the first and second carbon-ion RT. (E) A dose-volume histogram of this patient comprised of the first and second carbon-ion RT by using EQD2 conversion. [file Image_1.TIFF]

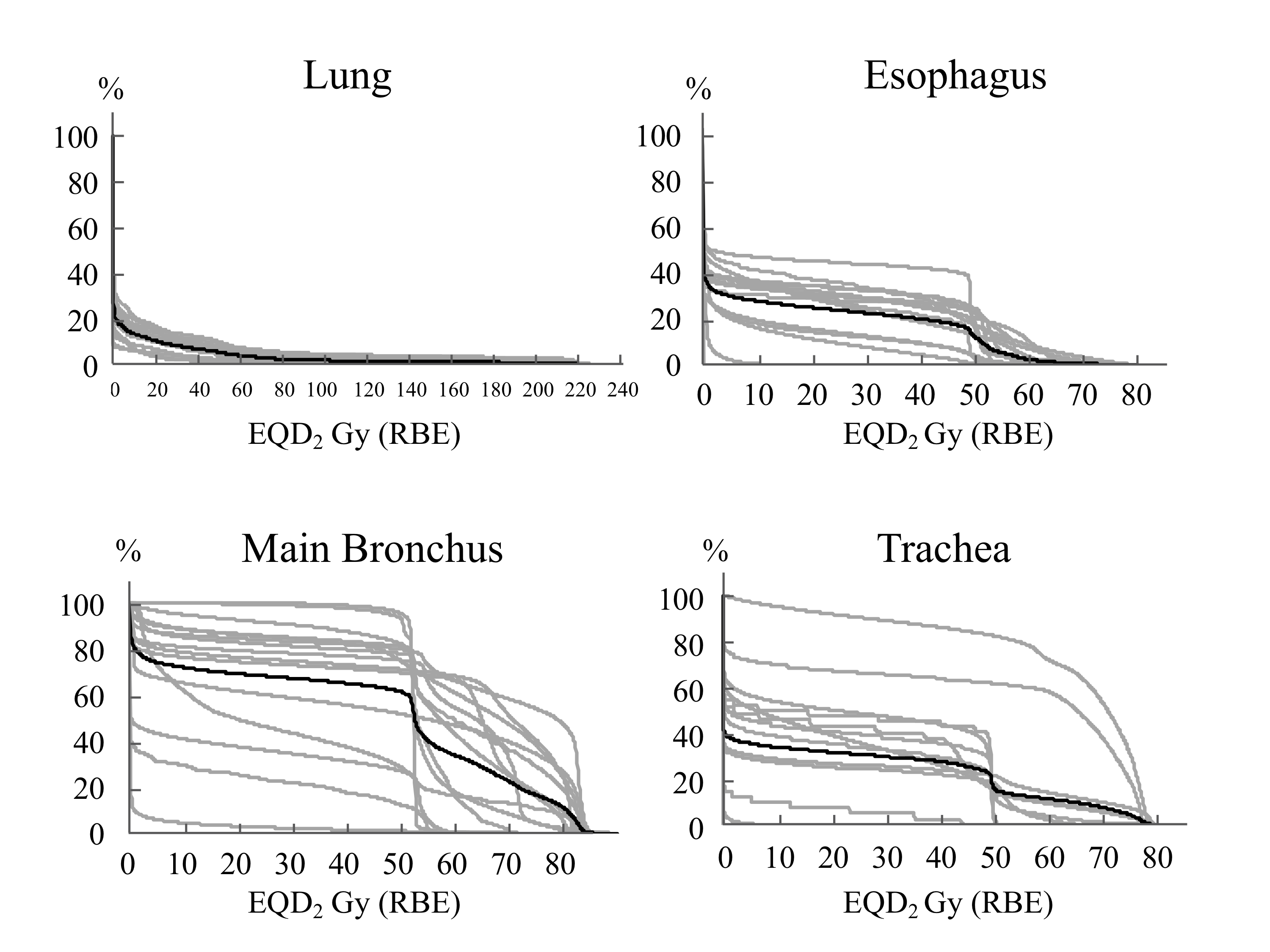

Supplement: Supplementary Figure 2 — Dose-volume histogram for the esophagus, lung, main bronchus, and trachea by using EQD2 conversion. The lines representing each patient are in gray, and the average is a black line. For patients that received carbon-ion radiotherapy (RT) as the first treatment, these histograms were composed of both the first and second RT. [file Image_2.TIFF]

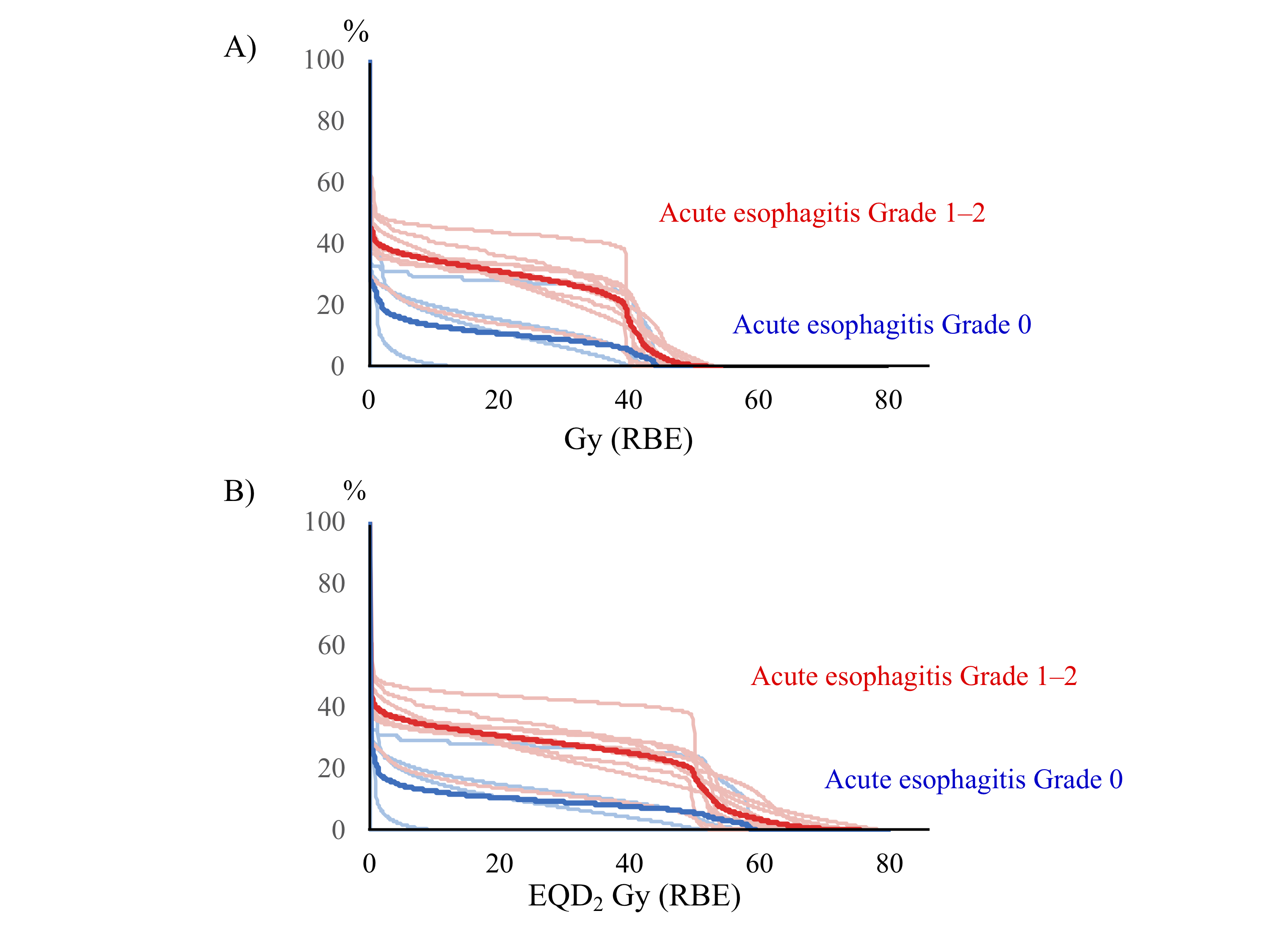

Supplement: Supplementary Figure 3 — (A) The dose-volume histogram parameters of 10 patients with acute esophagitis grade 1–2 (red line) and of 5 patients with grade 0 (blue line). Bold red and blue lines showed the average parameters of acute esophagitis grade 1–2 and grade 0, respectively. (B) The dose-volume histogram parameters were calculated by using EQD2 conversion. [file Image_3.TIFF]
