# Supplementary material for: Carbon-ion Radiotherapy for Isolated Lymph Node Metastasis After Surgery or Radiotherapy for Lung Cancer
Source: Front Oncol. 2019 Aug 7;9:731. doi: 10.3389/fonc.2019.00731 (PMC6692658; doi:10.3389/fonc.2019.00731)
Supplement: Supplementary Table 1 — A comparison of esophageal dose between acute esophagitis grade 0 and Grade 1–2. [file Table_1.docx]

**Supplementary Table 1.** A comparison of esophageal dose between acute esophagitis grade 0 and Grade 1–2.

| Acute adverse events | Grade 0 (n=10) | Grade 1–2 (n=5) | P-value |
| --- | --- | --- | --- |
| V20 Gy (RBE) | 11 .1 ± 11.9% | 31.3 ± 7.5% | < 0.01 |
| V30 Gy (RBE) | 9.0 ± 11.2% | 27.5 ± 8.1% | < 0.01 |
| V40 Gy (RBE) | 5.7 ± 10.3% | 15.6 ± 7.4% | 0.05 |
| V50 Gy (RBE) | 0 ± 0% | 0.58 ± 1.0% | 0.11 |
| Mean dose | 4.9 ± 4.8 Gy (RBE) | 13.0 ± 3.1 Gy (RBE) | < 0.01 |
| Maximum dose | 32.3 ± 21.8 Gy (RBE) | 49.4 ± 4.2 Gy (RBE) | 0.16 |
| Dose of 2 ml | 23.4 ± 20.2 Gy (RBE) | 43.2 ± 3.1 Gy (RBE) | 0.09 |

RBE: relative biological effectiveness
